# Treatment-resistant pediatric giant prolactinoma and multiple endocrine neoplasia type 1

**DOI:** 10.1186/s13633-015-0011-5

**Published:** 2015-07-15

**Authors:** Hoong-Wei Gan, Chloe Bulwer, Owase Jeelani, Michael Alan Levine, Márta Korbonits, Helen Alexandra Spoudeas

**Affiliations:** Section for Genetics and Epigenetics in Health and Disease, Genetics and Genomic Medicine Programme, University College London Institute of Child Health, 30 Guilford Street, London, WC1N 1EH UK; Section for Experimental & Personalized Medicine, Genetics & Genomic Medicine Programme, University College London Institute of Child Health, 30 Guilford Street, London, WC1N 1EH UK; The London Centre for Pediatric Endocrinology & Diabetes, Neuroendocrine Division, Great Ormond Street Hospital for Children NHS Foundation Trust, Great Ormond Street, London, WC1N 3JH UK; Department of Neurosurgery, Great Ormond Street Hospital for Children NHS Foundation Trust, Great Ormond Street, London, WC1N 3JH UK; Division of Endocrinology and Diabetes, The Children’s Hospital of Philadelphia, 34th and Civic Center Boulevard, Philadelphia, PA 19104 USA; Department of Pediatrics, University of Pennsylvania Perelman School of Medicine, 34th and Civic Center Boulevard, Philadelphia, PA 19104 USA; Centre for Endocrinology, Barts and the London School of Medicine & Dentistry, Queen Mary University of London, Charterhouse Square, London, EC1M 6BQ UK

**Keywords:** Familial prolactinoma, Macroprolactinoma, Pituitary neoplasms, Multiple endocrine neoplasia type 1, Survivorship

## Abstract

**Background:**

Pediatric pituitary adenomas are rare, accounting for <3 % of all childhood intracranial tumors, the majority of which are prolactinomas. Consequently, they are often misdiagnosed as other suprasellar masses such as craniopharyngiomas in this age group. Whilst guidelines exist for the treatment of adult prolactinomas, the management of childhood presentations of these benign tumors is less clear, particularly when dopamine agonist therapy fails. Given their rarity, childhood-onset pituitary adenomas are more likely to be associated with a variety of genetic syndromes, the commonest being multiple endocrine neoplasia type 1 (MEN-1).

**Case description:**

We present a case of an early-onset, treatment-resistant giant prolactinoma occurring in an 11-year-old peripubertal boy that was initially sensitive, but subsequently highly resistant to dopamine agonist therapy, ultimately requiring multiple surgical debulking procedures and proton beam irradiation. Our patient is now left with long-term tumor- and treatment-related neuroendocrine morbidities including blindness and panhypopituitarism. Only after multiple consultations and clinical data gained from 20-year-old medical records was a complex, intergenerationally consanguineous family history revealed, compatible with MEN-1, with a splice site mutation (c.784-9G > A) being eventually identified in intron 4 of the *MEN1* gene, potentially explaining the difficulties in management of this tumor. Genetic counseling and screening has now been offered to the wider family.

**Conclusions:**

This case emphasizes the need to consider pituitary adenomas in the differential diagnosis of all pediatric suprasellar tumors by careful endocrine assessment and measurement of at least a serum prolactin concentration. It also highlights the lack of evidence for the optimal management of pediatric drug-resistant prolactinomas. Finally, the case we describe demonstrates the importance of a detailed family history and the role of genetic testing for *MEN1* and *AIP* mutations in all cases of pediatric pituitary adenoma.

## Background

Pituitary adenomas account for <3 % of all childhood intracranial tumors with an estimated incidence of 0.1 cases/million/year [1, 2]. Prolactin (PRL)-secreting tumors alone account for 50-70 % of pituitary adenomas in children <20 years of age [3–5]. These are further classified into microprolactinomas (≤1 cm in maximum dimensions), macroprolactinomas (>1 cm) and giant prolactinomas (>4 cm with a serum PRL of >5300-10600 mU/l) [6, 7]. First-line therapy consists of dopamine agonists such as cabergoline, with surgery and radiotherapy being reserved for drug-resistant tumors or neuro-ophthalmological emergencies. Whilst 20-30 % of patients with known multiple endocrine neoplasia type 1 (MEN-1) develop prolactinomas [4, 8, 9], up to 6.5 % of sporadic pediatric prolactinomas are associated with previously undiagnosed MEN-1 [10].

We report a patient presenting to our quaternary pediatric neuroendocrine and oncology units with a treatment-resistant giant prolactinoma. His complex genetic history and challenging management illustrates the necessity for thorough history-taking, specialist multidisciplinary management and the potential for long-term neuroendocrine morbidity in this rare tumor.

## Case presentation

**Fig. 1:**
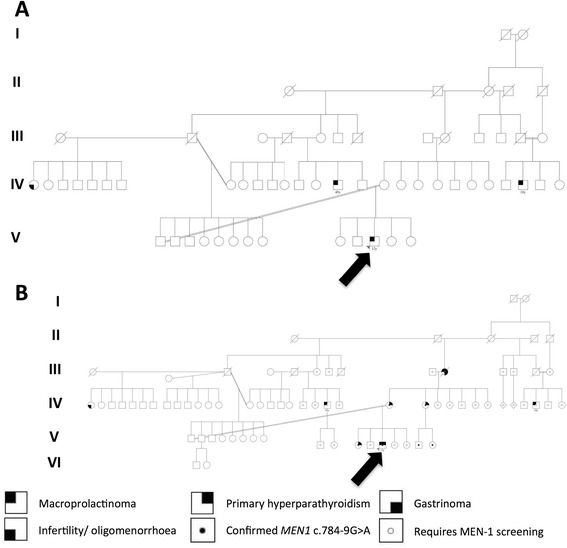
Patient family tree. Our patient’s family tree **a** at initial consultation and **b** at last follow-up after discovery of several other symptomatic and asymptomatic relatives who were subsequently confirmed to carry the same c.784-9G > A *MEN1* mutation as our proband

An 11-year-old Middle Eastern boy was referred to Great Ormond Street Hospital for Children with four years of headaches and 18 months of visual deterioration, previously investigated with a reportedly normal brain CT scan in his country of origin. There was no galactorrhea. At presentation he had a right-sided relative afferent pupillary defect, optic atrophy and could only perceive light with his right eye and count fingers with his left eye. Auxology was normal and he was in early puberty with testicular volumes of 4 mls bilaterally. Initial family history revealed an inter-generational multiply consanguineous family (Fig. 1a), with one maternal first half-cousin once removed treated with cabergoline at age 18 years and one paternal first half-cousin once removed undergoing transsphenoidal surgery at age 45 years, both for macroprolactinomas.

**Fig. 2:**
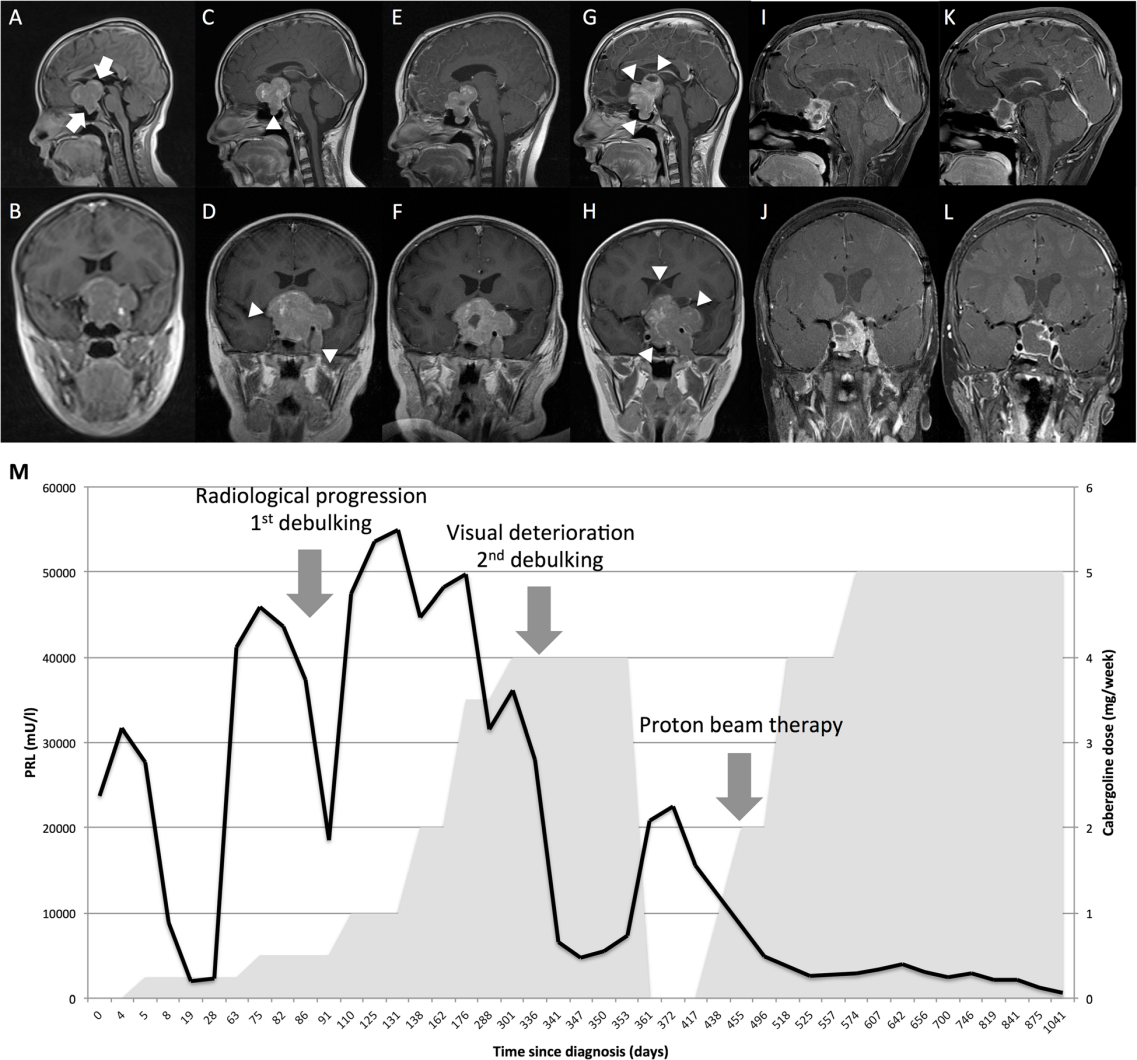
Patient MRI images and biochemistry results. Serial T_1_-weighted MRI images with gadolinium contrast demonstrating appearances of our patient’s giant prolactinoma **a**-**b** at diagnosis, **c**-**d** at first progression, **e**-**f** after first debulking, **g**-**h** at second progression, **i**-**j** after second debulking and radiotherapy, **k**-**l** at last follow-up. Arrows indicate tumor mass whilst arrowheads indicate sites of progression. **m** Serial prolactin concentrations (black line) and corresponding cabergoline dose (gray shaded area) in our patient since diagnosis illustrating multiple biochemical relapses despite multimodality treatment

Magnetic resonance imaging revealed a large, avidly-enhancing heterogeneous sellar and suprasellar mass merging with the pituitary gland, compressing the optic chiasm and encasing both internal carotid arteries with a maximal transverse diameter of 5.5 cm (Fig. 2a-b). A provisional radiological diagnosis of craniopharyngioma was made with a plan for surgical resection. However, biochemical testing revealed marked hyperprolactinemia (PRL 23723 mU/l, normal range 55-318) leading to the revised diagnosis of a giant prolactinoma. Dynamic testing revealed deficiencies in growth hormone (GH, peak to glucagon stimulation 1.1 ng/ml; insulin-like growth factor-1 (IGF-1) 196 ng/ml, normal range 143-693) and thyroid-stimulating hormone (TSH 3.7 mU/l, normal range <6.0; free thyroxine (fT_4_) 10.3 pmol/l, normal range 10.8-19.0) but normal hypothalamo-pituitary-adrenal (peak cortisol 516 nmol/l to synacthen stimulation) and -gonadal function (luteinizing hormone 0.3 U/l, follicle-stimulating hormone 0.9 U/l, testosterone 1.68 nmol/l).

Our patient was commenced on 250 μg/week cabergoline and levothyroxine supplementation. Despite an initial excellent PRL reduction in response to therapy, his tumor became extremely drug-resistant despite cabergoline dose escalation to 3.5 mg/week (Fig. 2m). Multiple relapses threatening vision required two transcranial interhemispheric debulking procedures and adjuvant proton beam irradiation, with the second debulking and radiotherapy undertaken at the Children’s Hospital of Philadelphia (CHOP, Fig. 2c-l). Tumor histology confirmed a prolactinoma with a high Ki67 index (11 %). During the second surgical debulking he experienced a preoperative anesthesia-related hemorrhagic stroke causing a transient left hemiparesis, and post-operatively lost all residual vision. He now has panhypopituitarism but has demonstrated declining PRL concentrations (500 mU/l) whilst maintained on 5 mg/week of cabergoline.

His complex family history alerted clinicians to the likelihood of an underlying genetic diagnosis but despite repeated consultations with multiple specialists (HAS, MK, OJ) no common ancestor linking all three macroprolactinoma cases could be found. Genetic testing of our patient for both *MEN1* and *AIP* mutations revealed a heterozygous *MEN1* intronic splice site mutation (c.784-9G > A). This has previously been shown to result in alternative splicing, a premature stop codon and a truncated, inactive MENIN protein [11–13].

Subsequent questioning revealed a history of renal stones and primary hyperparathyroidism in our patient’s mother and two maternal aunts, as well as a history consistent with MEN-1 (macroprolactinoma, primary hyperparathyroidism and gastrinoma) in his deceased maternal grandmother (Fig. 1b) who had been managed by our adult colleagues in the United Kingdom two decades earlier. Genetic testing confirmed the same mutation in his sister, mother, maternal grandmother, one maternal aunt and her two children, but not in his father or his paternal uncle. Most recently, our patient has developed primary hyperparathyroidism, and a parathyroidectomy is being planned. He continues to undergo regular screening including gut hormone profiling and echocardiograms at CHOP, all of which have been normal thus far. We are in the process of collaboratively tracing and screening other asymptomatic relatives in the wider family at risk.

## Discussion

The rarity of pediatric pituitary adenomas means that they are often not considered in the differential diagnosis of suprasellar tumors in this age group, leading to the potential misdiagnoses of craniopharyngioma or low-grade glioma, for which primary treatments are predominantly surgical, chemotherapeutic and/ or radiotherapeutic in nature. This case highlights the importance of a routine endocrine evaluation in all such cases, not only to document potential tumor-related pituitary dysfunction but also to identify functioning pituitary adenomas by measuring serum PRL, GH and adrenocorticotropic hormone, particularly as non-functioning pituitary adenomas are rare in children [14, 15, 5]. It also emphasizes that macroprolactinomas often present with non-endocrine raised intracranial pressure symptoms particularly in prepubertal males [16–19, 15]. It is important to distinguish true prolactinomas from hyperprolactinemia from other causes, but concentrations >5300 mU/l are usually diagnostic [7]. In all suspected cases, screening for macroprolactinemia [20], as well as serial serum dilution to exclude the hook effect – where extreme hyperprolactinemia causes falsely low assay results through interference of antibody-antigen complex formation [6, 21] – should always be performed.

Medical monotherapy with dopamine agonists is recommended as first-line treatment for all prolactinomas due to their excellent efficacy, likely preservation of residual pituitary function and low side-effect profile [7, 22]. Cabergoline in particular has an established record of being better-tolerated than bromocriptine with higher rates of resolution [7]. Cardiac valve regurgitation with high cumulative doses (4000 mg) has been described in Parkinson syndrome patients [23], but this is usually not reached with prolactinomas, as demonstrated by several adult studies [7, 24–27]. However, young patients such as ours commencing treatment earlier in life are more likely to reach this critical threshold, and at the current dose of 5 mg/week our patient will be exposed to 4000 mg of cabergoline within 16 years post-diagnosis, unless doses can be reduced in response to the efficacy of radiotherapy. Dose-safety profiles have additionally not been established in children, in whom toxicity even at moderate cabergoline doses may be increased. Quinagolide, a more recent non-ergot derived dopamine agonist is purported to have fewer side effects but to our knowledge has not been used in children [28].

The mechanism for development of drug-resistance is not well understood, and is thought to relate to dopamine D_2_ receptor downregulation [7]. MEN-1 is associated with larger tumors (84 % vs. 24 %) and treatment-resistance (56 % vs. 10 %) [29, 9]. Second-line management options for drug-resistant adult tumors include maximizing cabergoline dose to 11 mg/week, transsphenoidal or transcranial surgical resection, radiotherapy and the alkylating agent temozolomide [7, 6, 30, 31]. However, knowledge of the maximum safe therapeutic cabergoline dose, speed of dose escalation, long-term toxicities of temozolomide, and experience in pediatric transsphenoidal resections is limited. Consequently, optimal management of pediatric pituitary adenomas remains unclarified, suggesting the need for treatment in specialist neuroendocrine units with both adult and pediatric experience. Several authors report long-term tumor- and/ or treatment-related morbidities including hypopituitarism, obesity, dyslipidemia, infertility and reduced bone mineral density [16–18, 32, 6, 3].

9-22 % of pediatric pituitary adenomas are associated with genetic tumor-predisposing syndromes, the most well-known of which is MEN-1 with an estimated prevalence of 0.02-0.2 per 1000 [10, 29]. Whilst primary hyperparathyroidism is the commonest presenting manifestation, 17 % will present with a pituitary adenoma (classically a prolactinoma) [9]. As illustrated here, MEN-1 is an autosomal dominant condition characterized additionally by enteropancreatic tumors, non-functioning adrenocortical adenomas, angiofibromas, collagenomas, thyroid adenomas, meningiomas and other neuroendocrine tumors [8]. Other genetic endocrine syndromes associated with pituitary tumors are the familial isolated pituitary adenoma (FIPA) syndrome (of which *AIP* mutations are a subset), multiple endocrine neoplasia type 4 (*CDKN1B*), Carney complex (*PRKAR1A*), McCune-Albright syndrome (*GNAS*), SDH-related pituitary adenoma syndrome (*SDHB, SDHC, SDHD),* neurofibromatosis type 1 *(NF1)* and *DICER1* syndrome [33, 34, 4, 35–37].

Several authors strongly suggest testing for both *MEN1* and *AIP* germline mutations in all pediatric patients with PRL- and GH-secreting pituitary adenomas as they may be index cases for a given family [10, 33, 29]. A useful algorithm has been proposed by Korbonits *et al.* (2012). This is particularly important given that current guidelines recommend testing first-degree relatives of index cases and performing annual biochemical screening in carriers for insulinomas, PRL- and GH-secreting pituitary adenomas from age 5 years and for hyperparathyroidism from age 8 years [8].

## Conclusion

Pituitary adenomas must be considered in the differential diagnosis of all pediatric suprasellar masses, therefore mandating a detailed endocrine review and an accurate serum PRL measurement as management differs markedly from that of other tumors. Optimal management of dopamine agonist-resistant tumors in childhood remains unclear. A detailed family history of brain tumors and MEN-1-associated symptoms such as renal stones must be obtained and may require several consultations before being fully elucidated. We recommend that all children with PRL- or GH-secreting pituitary adenomas undergo *MEN1* and *AIP* mutation testing, with subsequent screening of first-degree relatives if found positive.

## Consent

Written informed consent was obtained from the parent of the patient for publication of this case report and any accompanying images. A copy of the written consent is available for review by the Editor-in-Chief of this journal.

## Abbreviations

CHOP: Children’s Hospital of Philadelphia
FIPA: Familial isolated pituitary adenoma (syndrome)
fT_4_: Free thyroxine
GH: Growth hormone
IGF-1: Insulin-like growth factor 1
MEN-1: Multiple endocrine neoplasia type 1
PRL: Prolactin
TSH: Thyroid-stimulating hormone
